# Effects of physical characteristics on metal–organic frameworks adsorption performance for water vapor: a review

**DOI:** 10.1039/d5ra10136k

**Published:** 2026-04-22

**Authors:** Wenxuan Zhang, Jinzhe Nie

**Affiliations:** a Beijing Key Lab of Heating, Gas Supply, Ventilating and Air Conditioning Engineering, School of Environmental and Energy Engineering, Beijing University of Civil Engineering and Architecture Beijing 100044 China niejinzhe_bucea@163.com

## Abstract

Metal–organic frameworks (MOFs), as one of the most rapidly advancing classes of porous materials in recent years, exhibit remarkable advantages in water vapor adsorption owing to their highly tunable pore structures, abundant surface active sites, and superior responsiveness to humidity. This review systematically investigates effects of key physical characteristics—including pore size, specific surface area, and pore volume—on the water vapor adsorption behavior of MOFs. By conducting a comparative analysis of performance data from nearly sixty representative MOFs, the study elucidates how distinct structural features govern adsorption mechanisms and thereby affect water uptake, such as chemisorption at low humidity, hydrogen-bonded molecular cluster formation within pores, and capillary condensation at medium to high humidity. The results demonstrate that microporous MOFs display characteristic step-shaped adsorption profiles and excellent cycling stability under low-humidity conditions, whereas mesoporous MOFs exhibit pronounced step-shaped adsorption accompanied by hysteresis loops at medium to high humidity. Research on large-pore MOFs remains limited due to challenges in maintaining structural stability. Increasing pore size generally enhances both specific surface area and pore volume, thereby enabling higher maximum saturation capacities. Furthermore, this review discusses the correlation between these physical characteristics and representative application scenarios—including indoor dehumidification and atmospheric water harvesting—providing theoretical insights and practical guidance for the rational design and optimization of MOFs.

## Abstract 2

MOFs capture water vapor *via* chemisorption at unsaturated metal sites, hydrogen bonding and cluster formation, and capillary condensation. Pore size determines the isotherm shape, while pore volume and surface area govern the maximum uptake.

## Graphical abstract

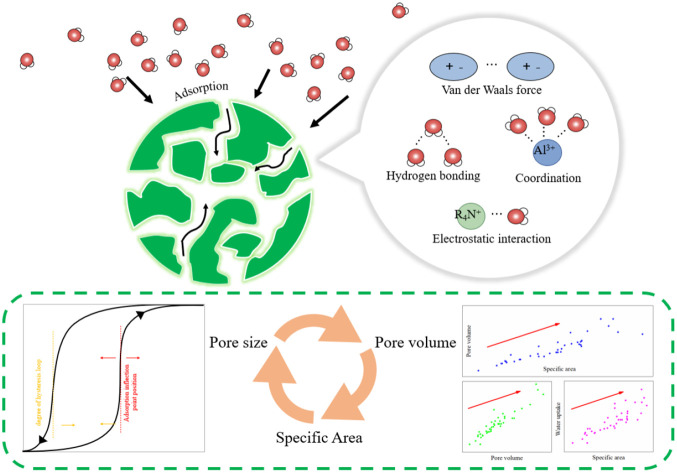

## 1. Introduction

Porous materials demonstrate considerable application potential in water vapor adsorption due to their abundant pore structures and high specific surface areas.^1–4^ These materials capture water molecules through physisorption or chemical interactions, enabling adsorption and regeneration under specific conditions, which makes them suitable for various environmental and energy systems.^2^ Their adsorption behavior is influenced by factors such as pore type, surface chemistry, and structural stability, leading to marked variations in adsorption capacity, response rate, and energy consumption among different materials.^5^ Porous materials can achieve efficient moisture capture and release with relatively low energy input, particularly in materials exhibiting a steep rise in their adsorption isotherms (S-shaped isotherms). The rapid response to water vapor in such materials offers promising solutions for diverse environmental and energy applications.^6,7^

Compared to traditional porous materials, MOFs, which have been developed for some time, demonstrate superior performance in both pore-related parameters and adsorption properties. Owing to their structural characteristics, MOFs exhibit highly tunable structures through various combinations of metal ions and organic linkers, as well as numerous modification strategies, rendering them exceptional in water vapor adsorption applications. Nearly 100 000 MOFs have been reported to date, finding extensive use in atmospheric water harvesting, heat transformation in mechanical systems, and water storage, gradually emerging as effective alternatives to conventional hygroscopic materials.^8^ Composed of metal ions or clusters coordinated with organic linkers, MOFs form highly ordered, open frameworks. Their highly tunable pore structures, abundant surface active sites, and outstanding adsorption response capabilities confer significant potential and unique advantages in the field of water vapor adsorption.^9–12^ For instance, Furukawa *et al.* systematically investigated the water stability and adsorption capacity of MOFs with different topologies, finding that Zr-based MOFs maintain excellent structural integrity under high humidity conditions.^13^ Kim *et al.* explored the water capture capability of MOF-801 under low humidity, proposing its potential for atmospheric water harvesting in arid regions.^6^

Based on water vapor adsorption mechanisms, MOFs have been widely applied in numerous fields, including humidity control in built environments, thermal transformation systems, atmospheric water harvesting, water storage and delivery, and gas separation and purification. MOF-303, integrated into water harvesting systems, enables low-energy dehumidification at ambient temperature, with a regeneration temperature significantly lower than that of traditional silica gel. It facilitates the formation of unsaturated water clusters within its cavities; during regeneration, these clusters aggregate and condense, substantially reducing energy consumption compared to conventional condensation methods, making this porous material highly suitable for water harvesting systems.^14^ In thermal transformation systems, csq-Zr-MOF-5 exhibited excellent cooling performance in adsorption heat pumps, with stable cycling performance and a coefficient of performance for heating as high as 0.88, approaching the theoretical limit.^15^ For indoor humidity control, MIL-101(Cr) demonstrates an extremely high water vapor adsorption capacity, 3–6 times greater than traditional desiccant materials, highlighting its superior potential for indoor dehumidification. However, the presence of potentially harmful metal clusters somewhat limits its application prospects.^16^ Some MOF composite materials, due to their thermal stability and resistance to hydrolysis in aqueous environments, can also be used in adsorption heat batteries. They utilize the adsorption and desorption of water vapor to release latent heat, enabling temporal energy management. Materials with ultra-high water adsorption capacities can also be applied for water storage.^17,18^ MOFs maintain selective adsorption capabilities even under humid conditions, making them suitable for CO_2_ capture and volatile organic compounds removal in moisture-laden environments.^19^ Whether for thermal transformation, indoor dehumidification, or other applications, the performance of water adsorption devices relies heavily on MOFs possessing superior and appropriate water vapor adsorption properties. The variation in performance among these porous materials is significantly influenced by synthesis methods, material types, and physical properties.

The performance of MOFs for water vapor adsorption is evaluated using a series of quantitative metrics, including maximum uptake, cycling stability, regeneration energy consumption, and adsorption profiles.^20^ To clearly represent the water adsorption performance under specific environmental conditions, the adsorption uptake curve at constant temperature but varying water vapor partial pressure is commonly used, termed the adsorption isotherm. Porous materials typically exhibit six types of adsorption isotherms, with the Type V (S-shaped) isotherm being highly characteristic of many MOFs.^21–23^ However, numerous crucial aspects of water adsorption performance cannot be directly deduced from the isotherm alone. The time required for the material to reach saturation uptake under operating conditions relates to its adsorption kinetics. The energy required for water desorption (regeneration energy), material susceptibility to hydrolysis, and performance after multiple cycles are not readily apparent from the isotherm and must be evaluated separately.

The practical application of MOFs in water adsorption is constrained by their material properties, which are intrinsically linked to their structural characteristics. The choice of metal ions and linkers, synthesis methods and processes directly impact the final physical structure of the material. As a subclass of porous materials, the adsorption performance of MOFs is profoundly influenced by the structural parameters of their pores.^5^ Key physical characteristics related to pores typically include specific surface area, pore size, and pore volume. These physical characteristics largely determine the water vapor adsorption characteristics of MOFs: pore size influences the mechanism of water molecule adsorption within the pores, specific surface area dictates the accessible adsorption interface for water molecules, and pore volume is closely related to the maximum adsorption capacity.^1,3,15,24,25^

Recently, Shi *et al.* comprehensively reviewed MOFs for water adsorption, emphasizing reticular chemistry strategies and device-level developments.^26^ Whereas their work provides a broad overview of MOF design, the present review adopts a complementary structure–property perspective, focusing on the quantitative effects of pore size, specific surface area, and pore volume on water vapor adsorption. Through critical analysis of data from nearly 60 MOFs (Table 1), the coupled influences of these parameters are elucidated and insights into optimizing physical attributes for targeted adsorption performance are provided, complementing the design principles outlined by Shi *et al.*

**Table 1:** Physical characteristics and water adsorption performance of reported MOFs

Materials	Pore size (nm)	Specific surface area (m^2^ g^−1^)	Pore volume (cm^3^ g^−1^)	*P*/*P*_0_ of the steep adsorption	Water uptake (g g^−1^(MOFs))	Ref.
MOF-303(Al)	0.6	—	0.54	0.1	0.48	64 and 65
MOF-841(Zr)	0.92	1390	0.53	0.26	0.5	13 and 66
MIL-53-FA(Al)	0.6	1080	0.49	0.2	0.53	67 and 68
CAU-6	0.91	760	0.34	—	0.3	13
CAU-10(Al)	0.7	635	0.43	0.15	0.36	69 and 70
MOF-74(Mg)	1.11	1250	0.53	0.05	0.75	13
MIL-160(Al)	0.5	1070	0.4	0.05	0.37	71
MIL-125	0.61	1510	0.68	—	0.3	72
CAU-23(Al)	0.76	1250	0.48	0.3	0.37	73
CUK-1(Co)	1.3	510	0.26	0.12	0.28	34
CUK-1(Mg)	—	580	0.28	0.23	0.36	34
CUK-1(Ni)	—	520	0.26	0.12	0.3	34
MIP-211(Al)	0.85	—	0.6	—	0.61	74
FDM-92(Al)	1.22	—	0.61	—	0.5	75
MOF-333(Al)	0.94	—	0.52	—	0.45	76
CAU-1(Al)	0.5/1.0	1300	0.55	0.38	0.55	63
CAU-1–NH2	—	1530	0.64	0.38	0.46	63
CAU-1–NHCOCH3	—	680	0.3	—	0.32	63
MOF-801(Zr)	0.48/0.56/0.77	990	0.45	0.05	0.36	13 and 77
UiO-66	0.74/0.84	1290	0.49	0.3	0.44	13
MIP-200(Zr)	1.3/0.68	1000	0.4	0.17	0.46	78
DUT-67	1.66/0.74	1560	0.6	—	0.5	79
HKUST-1	0.6/0.9	1340	0.62	0.05	0.5	80
ALP-MOF-1	1.2/0.95	1900	—	—	0.6	81
ALP-MOF-2	1.2/0.95	1860	—	—	0.38	81
MOF-804	0.72/0.68	1145	0.46	0.35	0.41	13
MOF-805	0.95/0.86	1230	0.48	0.28	0.4	13
SIM-1(Zn)	0.65	570	0.3	—	0.14	45
ZIF-8	1.16	1000	0.6	0.8	0.02	82
Kag-MOF-1	0.38	210	0.12	—	0.14	83
MOF-802	0.56	<20	<0.01	—	0.11	13
BUT-46A(Zr)	1.6/3.5	1550	0.69	0.44	0.52	84
BUT-46B(Zr)	1.6/3.5	1430	0.65	0.51	0.5	84
BUT-46F(Zr)	1.6/3.5	1563	0.71	0.4	0.6	84
BUT-46W(Zr)	1.6/3.5	1565	0.71	0.27	0.63	84
Ni-BPP	1.7	2039	0.88	0.1	0.72	85
Ni-TPP	2.3	1975	1.14	0.26	0.84	85
MOF-808	1.84	2360	0.84	0.28	0.6	13
PIZOF-2(Zr)	2	1250	0.68	0.7	0.68	13 and 62
Co2Cl2(BTDD)	2.2	1912	—	0.28	0.97	35
Ni2Cl2(BTDD)	2.2	1752	—	0.3	0.77	35
MIL-100(Al)	2.5/2.9	1814	1.14	0.3	0.5	37
MIL-100(Fe)	2.5/2.9	1917	1	0.3	0.77	37 and 86
MIL-100(Cr)	2.5/2.9	1517	—	0.3	0.8	87
MIL-101(Cr)	2.6/3.4	5900	2	0.4	1.6	71,88 and 89
Cr-soc-MOF-1	1.7	4549	2.1	0.6	1.95	90
DUT-68(Zr)	0.8/1.3/1.4/2.8	891	0.41	0.4	0.34	41
DUT-68(Hf)	—	749	0.34	0.38	0.29	41
DUT-69(Hf)	—	450	0.22	—	0.2	41
DUT-69(Zr)	—	560	0.31	—	0.26	41
ISO-NU-1000(Zr)	1.3/3.2	—	1.27	—	1.1	91
Facac-NU-1000(Zr)	1.2/2.7	—	1.36	—	1.02	92
ZJNU-30(Zr)	2.1/1.4/0.7	3116	1.24	—	1.2	66
UiO-67	2.16/1.1	1639	0.9	—	0.2	93
BIT-66	0.65/2.58	1417	0.87	0.71	0.6	94
Y-shp-MOF-5	1.2	1550	0.63	0.45	0.55	95

Although numerous studies have reported on the application, performance, and related physical properties of MOFs for water vapor adsorption, much of this research focuses on specific material types or application scenarios. A systematic investigation into how the physical properties of MOFs influence their adsorption performance and practical application suitability remains lacking. Therefore, this review aims to systematically elucidate the influence of material physical characteristics on water vapor adsorption and desorption behavior. Furthermore, it explores the correlation between water adsorption performance under different physical properties and their suitability for typical application scenarios.

## 2. Water adsorption in MOFs

As porous materials, MOFs share the fundamental structural characteristics of this class: abundant pore types and a high density of pores enable them to host a large number of water molecules and other gas species. They capture target molecules on the pore surfaces through physisorption or chemical interactions, thereby achieving the adsorption effect. However, MOFs differ from traditional porous materials. Their superior adsorption performance is attributable to their structural composition and unique properties. Before discussing the influence of their physical characteristics on water adsorption, it is necessary to detail the definition and material properties of MOFs, as well as the methods used to evaluate their adsorption performance for water vapor.

### 2.1 Metal–organic frameworks

In contrast to traditional porous materials, which are primarily composed of inorganic frameworks or carbon-based structures, MOFs are a class of crystalline porous materials formed by the self-assembly of metal ions/clusters and organic linkers *via* coordination bonds.^10,27,28^ This hybrid inorganic–organic composition endows MOFs with a high degree of designability: metal nodes determine the framework's stability and affinity, while organic linkers modulate pore size and surface chemical environment. Furthermore, solvents used in the synthesis process can influence the final topology and physical characteristics.^29,30^ Consequently, MOFs exhibit tunability in pore size distribution, specific surface area, and surface functionalization far exceeding those of traditional materials, providing unique advantages for their application in water vapor adsorption and dehumidification.^5,12,31^

#### 2.1.1 Compositions

The fundamental components of MOFs include metal ions (or metal clusters), organic ligands, and solvents potentially involved in the synthesis process. The judicious selection and combination of these three components not only determine the topology and physical characteristics of MOFs but also directly influence their water vapor adsorption performance and stability.

##### 2.1.1.1. Metal ions

Metal ions serve as the nodal units of MOFs, typically sourced from inorganic metal salts. Metal ions with different valences impart varying stability and hydrophilicity to the framework. Divalent metal ions (*e.g.*, Cu^2+^, Zn^2+^, Ni^2+^) are commonly used to construct classic MOFs such as HKUST-1 and ZIF series.^32–36^ Trivalent metal ions (*e.g.*, Fe^3+^, Al^3+^, Cr^3+^) often enhance hydrothermal stability due to their stronger coordination ability.^37,38^ Tetravalent metal ions (*e.g.*, Zr^4+^, Hf^4+^, Ti^4+^) are widely employed in the synthesis of highly stable MOFs such as UiO-66 and MIL-140.^39–41^ In recent years, lanthanide elements (*e.g.*, Ce, Eu) and p-block elements (*e.g.*, Ga, In) have also been introduced into MOF design to expand their structural diversity and functionality.^42–45^

##### 2.1.1.2. Organic ligands (linkers)

Organic ligands (linkers), featuring carboxylate, imidazole, or other multidentate coordinating groups, connect with metal ions to form three-dimensional frameworks. The length, rigidity, and degree of functionalization of the ligands determine the pore size, specific surface area, and hydrophilic/hydrophobic characteristics of the pore channels.^46^ Common examples include terephthalic acid, 1,3,5-benzenetricarboxylic acid, fumaric acid, and thiophenedicarboxylate.^47,48^ Theoretically, longer ligands can yield larger specific surface areas and pore volumes, thereby enhancing adsorption capacity.^49^ Introducing polar functional groups (*e.g.*, –NH_2_, –OH) onto the ligands can strengthen the affinity for water molecules and modulate the shape of the adsorption isotherm.^50,51^

##### 2.1.1.3. Solvents

Solvents in MOF synthesis act not only as reaction media but may also function as structure-directing agents or guest molecules participating in framework formation. The polarity, coordination ability, and volatility of the solvent significantly influence the final topology and physical characteristics of MOFs. For instance, solvents like *N*,*N*-dimethylformamide and *N*,*N*-diethylformamide are commonly used in solvothermal synthesis, but their toxicity and environmental concerns limit large-scale application.^52^ In recent years, green solvents such as water, ethanol, and ethyl acetate have gained increasing attention.^53,54^ Furthermore, solvent molecules remaining in the pores after synthesis must be removed *via* an activation process to release the pore space and restore the adsorption activity of the metal sites.

#### 2.1.2. Intrinsic properties

The exceptional performance of MOFs stems not only from the diversity of their constituent elements but also from their unique intrinsic properties. These properties determine their affinity for water vapor, the shape of their adsorption isotherms, and their cycling stability, thereby directly influencing their performance in applications such as dehumidification, thermal transformation, and atmospheric water harvesting.^12^ The intrinsic properties of MOFs are primarily manifested in metal sites, ligand functionalization and structural expansion, as well as porosity and specific surface area.

MOFs often contain a large number of coordinatively unsaturated sites(CUS). These sites are typically occupied by solvent or guest molecules after synthesis and require removal *via* an activation process, such as heating under vacuum or solvent exchange, to expose the highly polar metal nodes. The exposed metal sites can form strong interactions with water molecules, enabling MOFs to exhibit measurable adsorption capacity even at low relative humidity(RH).^13^ The density and recoverability of these CUS directly govern the low-humidity adsorption performance of MOFs, making the regulation of metal nodes crucial in material design.^55^

Beyond metal sites, the functionalization of organic ligands provides an effective pathway for tuning the hydrophilicity and hydrophobicity of MOFs. Introducing polar groups (*e.g.*, –NH_2_, –OH, –NO_2_) onto the ligands can enhance the framework's affinity for water molecules, altering the trigger point (*P*/*P*_0_) and shape of the adsorption isotherm, thus enabling precise control over adsorption behavior.^50,51^ However, functionalization is often accompanied by a reduction in specific surface area or pore volume,^3^ necessitating a balance between hydrophilicity and porous structure. Furthermore, the topology of MOFs is highly extensible. Employing elongated ligands or multidentate linkers can significantly increase pore volume and specific surface area while maintaining structural consistency.^56^ Composites of MOFs with inorganic salts or carbon-based materials have also proven effective in improving heat and mass transfer properties, thereby enhancing cycling stability and adsorption rates.^57–59^

The topological structure of MOFs, determined jointly by metal nodes and organic ligands, endows them with highly tunable physical characteristics. Benefiting from the flexible choice of metal ions and ligands, the specific surface area of MOFs can range from hundreds to thousands of square meter per gram, with porosity up to 90% and pore sizes spanning the microporous and even sub-nanometer scales.^10,60^ This highly controllable pore structure allows MOFs to achieve suitable adsorption performance across different humidity ranges: microporous structures favor strong adsorption at low humidity, while mesoporous or hierarchical pore structures help increase the maximum water uptake and working capacity.^61^ Additionally, synthesis conditions can significantly influence the pore size distribution and specific surface area, thereby providing additional means for optimizing MOF performance.^27^

#### 2.1.3. Pore size, specific surface area and pore volume

MOFs are constructed from metal ion nodes and organic linker units. The assembly of these components typically generates a significant volume of void space, which can be conceptualized as a multitude of internal pores, thereby classifying MOFs as porous materials. For any porous material, the size, geometry, and overall architecture of its pores are decisive factors influencing its macroscopic properties. The vast diversity of available metal ions and organic linkers endows MOFs with exceptional structural flexibility. Their pores are predominantly of molecular dimensions (ultramicropores and micropores), and they exhibit an extremely wide range of accessible specific surface areas and pore volumes. Furthermore, the synthesis methods and reaction conditions significantly impact the final physical characteristics. Extensive research has consistently demonstrated that the physical properties of these pores profoundly influence the outcomes of gas adsorption within them.

#### 2.1.4 Influence of synthesis methods on physical properties and adsorption behavior

The synthesis conditions of MOFs significantly influence their final physical characteristics, including pore size distribution, defect density, crystallinity, and particle morphology, which in turn determine water vapor adsorption performance. Three representative synthesis approaches are discussed below.

Solvothermal synthesis is the most widely used method for MOF preparation. By adjusting parameters such as temperature, reaction time, solvent composition, and template additives, researchers can control crystal nucleation and growth rates, thereby tuning crystal size, morphology, and defect density.^38^ For instance, UiO-66 synthesized at higher temperatures or with extended reaction times typically exhibits larger crystallites and fewer defects, resulting in more uniform pore structures and enhanced hydrolytic stability.^55^ Conversely, rapid crystallization under milder conditions often introduces structural defects that can create additional open metal sites, potentially improving low-humidity water uptake at the expense of long-term cycling stability.

Microwave-assisted synthesis offers rapid and uniform heating, leading to accelerated nucleation and significantly reduced crystallization times compared to conventional solvothermal methods. This approach typically produces smaller crystallites with higher surface areas and, in some cases, increased defect densities due to the rapid formation kinetics.^36^ For example, microwave-synthesized MIL-100(Fe) exhibits faster water adsorption kinetics and comparable or higher uptake capacities than its conventionally synthesized counterpart, making it attractive for applications requiring rapid cycle times.^62^

Modulated synthesis employs coordinating modulators (*e.g.*, acetic acid, benzoic acid, trifluoroacetic acid) that compete with organic linkers for coordination to metal nodes. By slowing down the crystallization process, modulators enable the formation of larger, more perfectly ordered crystals with controlled defect concentrations. This strategy is particularly effective for Zr-based MOFs such as UiO-66 and NU-1000, where modulator concentration directly influences pore size distribution, surface area, and water stability.^38,63^ Optimal modulator content can reduce defect-related pore heterogeneity while maintaining high porosity, leading to improved water adsorption performance and cycling stability.

The choice of synthesis method therefore provides an additional lever for tailoring MOF properties. While solvothermal synthesis offers versatility and scalability, microwave methods enable rapid production with enhanced kinetics, and modulated synthesis allows precise control over defect chemistry. Understanding these synthesis-structure-performance relationships is essential for developing MOFs with optimized water vapor adsorption characteristics.

### 2.2 Water adsorption

**Fig. 1:**
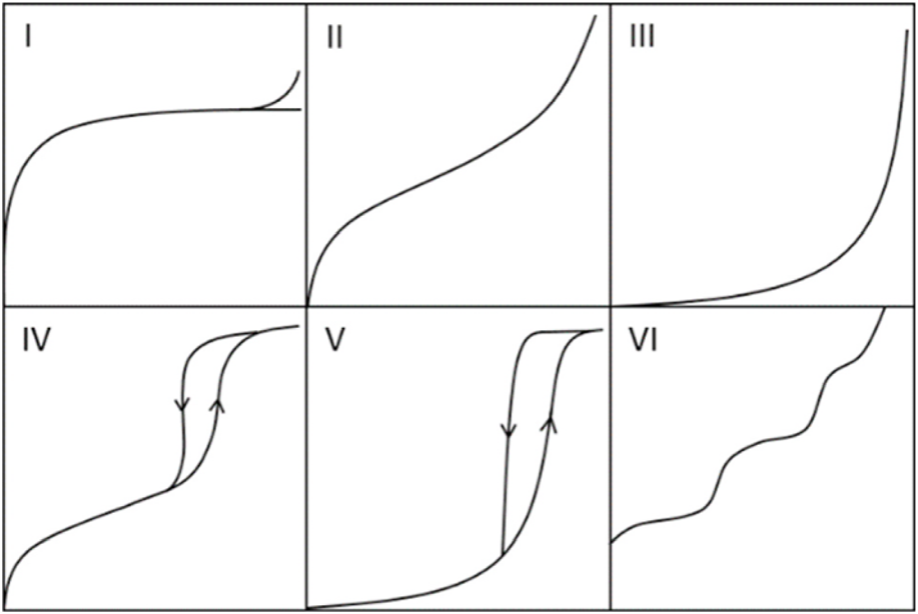
Classification of isotherms based on IUPAC, reproduced from ref. 27 with permission from Walter de Gruyter Berlin/Boston,^27^ Copyright 2016.

To graphically represent the water vapor adsorption characteristics of materials, the International Union of Pure and Applied Chemistry (IUPAC) classifies the adsorption curves of gases or water vapor into six types of adsorption isotherms (Fig. 1).^27^ These isotherms serve as crucial tools for characterizing a material's water uptake capacity and its affinity for water vapor. The curves visually depict the saturation uptake capacity of a material for moisture at different pressures. Furthermore, the shape and progression of the isotherm allow for a general assessment of the material's affinity towards water molecules.

Type I: characterized by a sharp increase in uptake at low relative pressure, followed by a plateau. It is typical for monolayer adsorption in non-porous or microporous materials.

Type II: exhibits a gradual increase in adsorption capacity with rising pressure, representing multilayer adsorption on non-porous or macroporous surfaces.

Type III: displays a concave shape relative to the pressure axis, indicating weak interactions between the adsorbate and adsorbent.

Type IV: features a steep uptake at a certain pressure, where capillary condensation becomes dominant, often accompanied by a distinct hysteresis loop.

Type V: shows a sharp increase in adsorption at high relative pressures, typical for adsorption behavior in large-pore materials, often associated with weak adsorbent–adsorbate interactions.

Type VI: exhibits step-wise increases in adsorption, usually indicative of complex pore structures or the presence of multiple distinct adsorption sites.

Most MOFs exhibit step-shaped water vapor adsorption behavior, particularly those with a certain degree of hydrophilicity. Their adsorption profiles typically correspond to Type I, II, or IV isotherms. When evaluating the water adsorption performance of these MOFs, focus is often placed on the maximum saturation uptake capacity from the isotherm, the position of the inflection point in the steep adsorption, and the presence or absence of a hysteresis loop during desorption.

However, crucial performance aspects for practical application cannot be fully captured by the isotherm alone. For instance, MOFs with a Type I isotherm, due to their strong hydrophilicity, often require more stringent regeneration conditions. Materials exhibiting a Type III isotherm may show significant uptake only at very high pressures, but their performance remains poor across a broad pressure range; such materials are still generally considered hydrophobic. Similarly, many other critical adsorption characteristics—including adsorption kinetics, cyclic performance, and long-term stability—cannot be directly derived from the equilibrium isotherm data.

## 3. Effects of physical characteristics on water adsorption behavior of MOFs

The interconnected units comprising metal ions and organic linkers in metal–organic frameworks inherently contain abundant porosity. Variations in the metal ions, linkers, topological structures, and synthesis methods all influence the adsorption mechanism of adsorbates within the pores. From the perspective of topology, as porous materials, the physical properties related to the pores in MOFs—specifically, key parameters such as specific surface area, pore size, and pore volume—directly dictate the adsorption performance for target molecules. This section systematically reviews nearly 60 MOF materials, spanning microporous to mesoporous regimes, to elucidate the influence of specific surface area (ranging from hundreds to several thousands of square meter per gram) and pore volume on water vapor adsorption performance, with a particular focus on the governing trends for maximum saturation uptake capacity.

### 3.1 Pore size

Based on the unique structural characteristics of MOFs, the adsorption mechanisms of water molecules can be broadly categorized into three types: (i) chemisorption between CUS and water molecules; (ii) formation of molecular clusters *via* physical interactions such as hydrogen bonding within the pores; (iii) capillary condensation. MOFs with different pore size ranges influence the ultimate water adsorption performance through different dominant adsorption mechanisms.^96–98^ The water vapor adsorption behavior, manifested in the performance data of adsorption isotherms, is governed by the predominant mechanism, which varies from microporous to mesoporous materials.

Typically, microporous (<2 nm) MOFs exhibit significant water uptake at low humidity. Those displaying S-shaped (Type V) isotherms have their adsorption inflection points at lower relative pressures. Hydrophilic microporous MOFs perform well in the low-humidity range, often approaching their maximum saturation capacity within the medium humidity range (30%∼60% RH). However, their total water uptake is generally lower than that of hydrophilic materials with larger pores. Furthermore, the strong chemical interaction between CUS and water molecules makes desorption more difficult for some highly hydrophilic microporous materials, necessitating more stringent conditions for regeneration.^31^ The adsorption process at these unsaturated sites is generally reversible, typically showing no hysteresis loop on the isotherm. This reversibility also ensures good cycling performance for water vapor adsorption.^71^

**Fig. 2:**
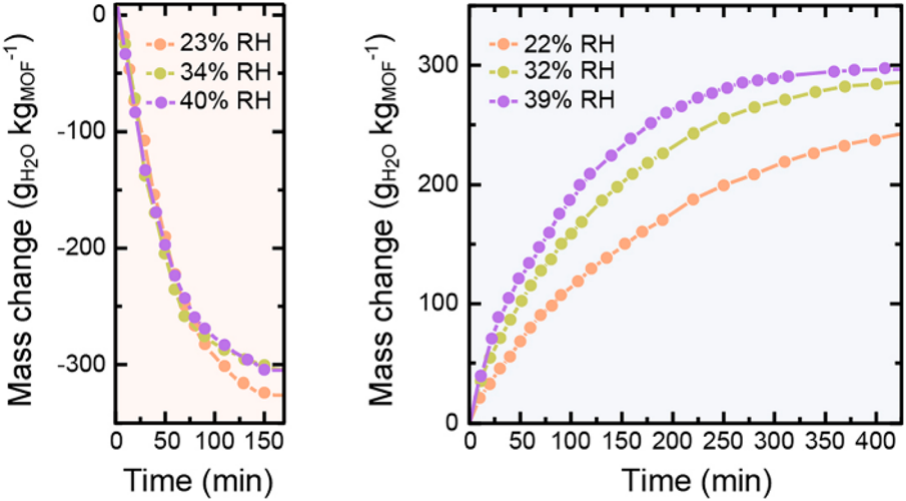
Water adsorption (right) and desorption (left) kinetics of MOF-303 at various RH, reproduced from ref. 68 with permission from American Chemical Society,^68^ copyright 2019.

**Fig. 3:**
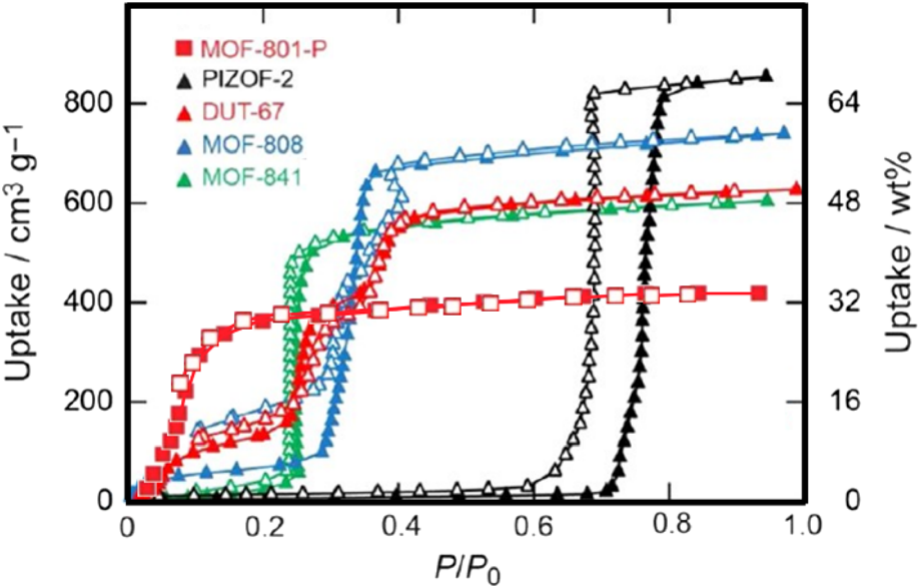
Water adsorption isotherms of MOF-841, MOF-801-P, and various other porous materials, reproduced from ref. 13 with permission from American Chemical Society,^13^ copyright 2014.

Due to their pronounced hydrophilicity, microporous MOFs have been extensively studied for water vapor adsorption. These hydrophilic materials exhibit characteristic microporous water adsorption behavior. MOF-303, noted for its water adsorption response at low humidity, was utilized by Hanikel *et al.* in an active atmospheric water harvesting device. It begins significant water uptake at around 10% RH, approaching its maximum capacity by approximately 20% RH, and desorbs rapidly under conditions of 85 °C and 0% RH. Compared to other materials in that study, such as Al-fumarate, MOF-303 possesses a more suitable adsorption inflection point and a larger uptake capacity (Fig. 5). Additionally, MOF-303 shows almost no hysteresis loop and no loss of adsorption capacity after multiple adsorption–desorption cycles. These advantages are attributed to its hydrophilic ligands and the topology based on aluminum clusters, which also confers excellent hydrolytic stability. Furthermore, its fast adsorption and desorption rates (Fig. 2) make it suitable for application in active water harvesting devices leveraging its superior dynamic adsorption properties.^65^ In contrast, MOF-841 also exhibits steep adsorption behavior within a similarly low humidity range.^13^ However, due to its larger pore size, the adsorption inflection point on its water vapor isotherm shifts to a higher relative pressure (starting around 20% RH), yet it still approaches its maximum capacity below 30% RH. In the study by Furukawa *et al.*,^13^ comparing it with materials like MOF-808 and PIZOF-2 (Fig. 3), the smaller-pore-sized MOF-841 showed an earlier inflection point. DUT-67, featuring a dual-pore-size system, exhibited multiple steps in its isotherm. Both MOF-841 and DUT-67 showed no hysteresis loops. Conversely, PIZOF-2, due to its hydrophobicity and larger pores, had its adsorption inflection point at a high relative pressure (70% RH) and displayed a hysteresis loop. After multiple cycling tests, MOF-841 maintained consistent water uptake. MOF-801-P, with even smaller pores than MOF-841 and stronger hydrophilicity (resulting in a more Type I-like isotherm), also maintained strong cycling stability, but its maximum adsorption capacity was significantly lower than that of MOF-841.

The adsorption characteristics of microporous materials featuring multiple pore sizes are more complex. While materials with hierarchically sized pores can sometimes exhibit the distinct adsorption properties of each pore level in their isotherms, others may only display the characteristics of one dominant pore type. CAU-1(Al) was initially synthesized by Stock *et al.* and possesses a structure containing two types of cage structures: 1 nm octahedral cages and 0.5 nm tetrahedral cages.^63^ Despite having these two types of micropores, CAU-1(Al) exhibits water vapor adsorption behavior typical of microporous materials. Although its adsorption inflection point is located in the medium humidity region (around 35% RH), it still shows some adsorption capacity at low humidity. However, due to its relatively poor cycling stability and comparatively low maximum saturation capacity, it is rarely considered a primary candidate for water adsorption applications.

Following functionalization with hydrophobic groups, the humidity range at which the steep adsorption uptake occurs in CAU-1 remains unchanged. However, this functionalization, involving the introduction of functional groups capable of hydrogen bonding and the consequent reduction in effective pore size, leads to a decrease in the overall specific surface area and pore volume. Consequently, both CAU-1–NH_2_ and CAU-1–NHCOCH_3_ show a significant reduction in maximum saturation uptake capacity and also exhibit slight hysteresis loops (Fig. 6). Similarly, BIT-66, which also features a dual-pore system, demonstrates analogous adsorption behavior. Its structure comprises micropores (0.65 nm) and larger mesopores (2.58 nm).^94^ Adsorption occurs in the low-humidity region *via* the micropores and open metal sites. The inflection point observed in the medium-humidity region is primarily attributed to capillary condensation within the larger mesopores and is accompanied by a distinct hysteresis loop. A stable adsorption capacity is only achieved after multiple cycles. This poor cycling stability is attributed to the different desorption behaviors of water molecules residing in the different pore types: water within the micropores is more difficult to remove, and incomplete desorption from these micropores negatively impacts the adsorption behavior in subsequent cycles.

**Fig. 4:**
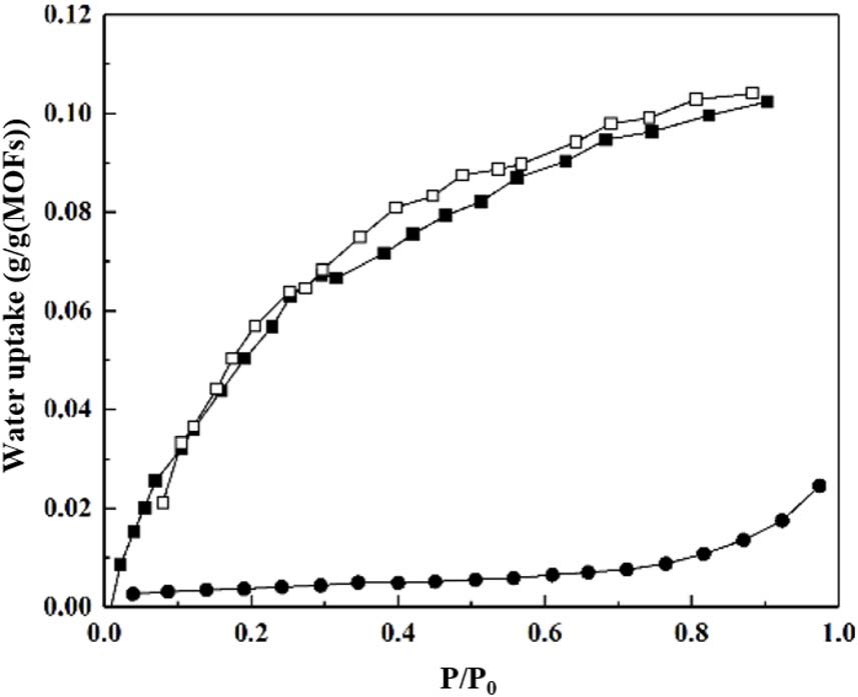
Water adsorption isotherms of MOF-802(■ = adsorption, □ = desorption), reproduced from ref. 13 with permission from American Chemical Society,^13^ copyright 2014, and ZIF-8(● = adsorption), reproduced from ref. 85 with permission from Advanced Functional Materials published by Wiley-VCH GmbH,^85^ copyright 2020.

Hydrophobic materials within the microporous range typically exhibit Type VI or Type I-like isotherms. The former, characterized by relatively larger pores, can achieve some water uptake at high humidity *via* capillary condensation, while the latter generally exhibits negligible water vapor adsorption capacity (Fig. 4).

**Fig. 5:**
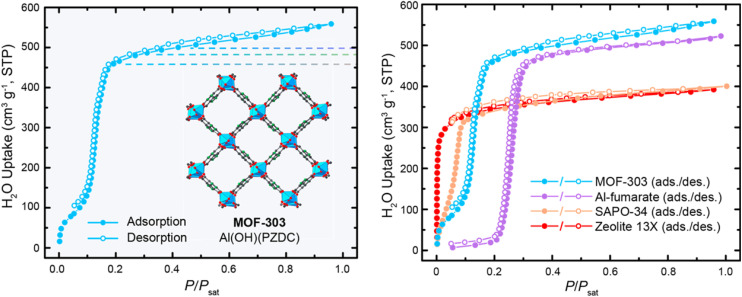
Water adsorption isotherms of MOF-303, UiO-66, and other porous materials, reproduced from ref. 68 with permission from American Chemical Society,^68^ copyright 2019.

**Fig. 6:**
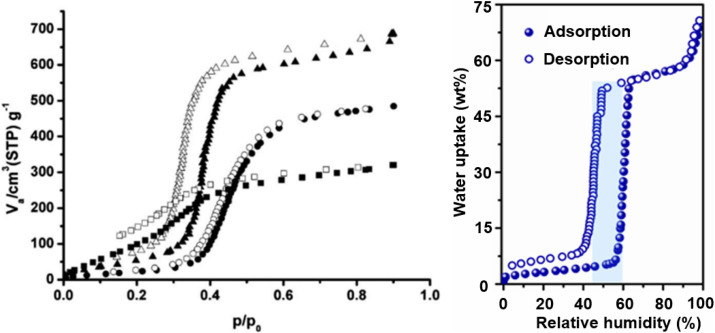
(Left) water adsorption isotherms of functionalized CAU-1-X (–NH2: ● = adsorption, ○ = desorption; –NHCH3: ▲ = adsorption, △ = desorption; –NHCOCH3: ■ = adsorption,□ = desorption), reproduced from ref. 63 with permission from Royal Society of Chemistry,^63^ copyright 2012; (Right) adsorption isotherms of BIT-66, reproduced from ref. 97 with permission from Wiley-VCH Verlag GmbH & Co. KGaA, Weinheim,^97^ copyright 2019.

ZIF-8 demonstrates extreme hydrophobicity in water vapor adsorption measurements: it shows almost no equilibrium uptake at low and medium humidity, with a sharp adsorption step occurring only at very high relative pressures.^82,99^ This step, attributed to capillary condensation, results in a very low total water uptake. Škrjanc *et al.* reported an adsorption capacity of approximately 0.034 g g^−1^(MOFs) for ZIF-8 at a high RH (70% RH), while Logan *et al.* measured a cyclable working water capacity of < 6 g kg^−1^(MOFs).^100,101^ Similarly, MOF-802 exhibits a Type I-like isotherm. As a hydrophobic microporous material, it also possesses a very small specific surface area and pore volume. Its maximum saturation water vapor uptake capacity is slightly higher than that of ZIF-8, reaching about 0.1 g g^−1^(MOFs) (Fig. 4, 5 and 6).

Mesoporous MOFs (2–50 nm), particularly hydrophilic variants, predominantly exhibit S-shaped (Type V) isotherms. Their larger pore sizes provide more adsorption sites and greater volume to accommodate water molecules, while also differentiating their adsorption mechanism from microporous MOFs. Hydrophilic mesoporous MOFs typically display their steep adsorption step in the medium humidity region. They show some uptake at low humidity *via* hydrophilic sites or functional groups, but the majority of water adsorption occurs in the medium humidity range. This stepwise uptake is usually induced by capillary condensation within the larger pores, which becomes the dominant adsorption mechanism in mesoporous materials and is often accompanied by a pronounced hysteresis loop.

Among hydrophilic mesoporous MOFs, the water vapor adsorption behavior of the MIL-100 and MIL-101 series is highly representative. The MIL-100 series most commonly comprises three materials formed from Fe, Al, or Cr metals with the BTC linker. They share similar physical properties, such as pore size and specific surface area, and contain pores of microporous dimensions (about 0.65 nm). MIL-100(Fe) and MIL-100(Al) exhibit two distinct inflection points in their water vapor adsorption isotherms. Initial adsorption at low humidity occurs *via* water molecules binding to CUS, followed by two steep uptake steps (around 25% RH and 35% RH). This can be interpreted as the sequential filling of pores of two different sizes: micropore filling associated with unsaturated sites at low humidity, and mesopore filling *via* capillary condensation of water at medium humidity. MIL-100(Cr) and MIL-100(Fe) approach their maximum saturation capacity around 45% RH, which is significantly higher than that of MIL-100(Al).^37,86,87^

**Fig. 7:**
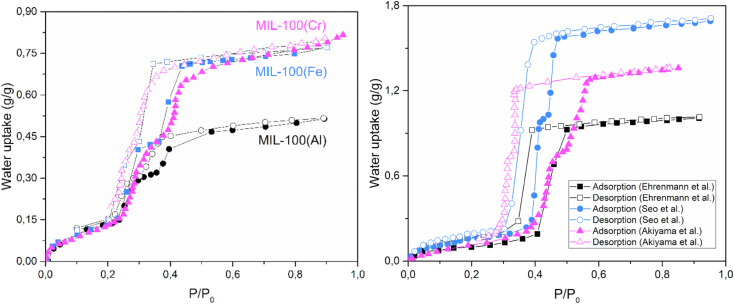
Water adsorption isotherms of the MIL-100 series^37,89,90^ and of MIL-101(Cr)^92,102,103^ synthesized by various research groups, reproduced from ref. 3 with permission from Elsevier,^3^ copyright 2020.

Subsequent research reported on the MIL-101 series. Due to hydrolysis issues, studies on the water vapor adsorption of the Fe- and Al-based MIL-101 are relatively scarce, while the Cr-based variant shows more prominent performance. Although MIL-101(Cr) possesses a pore size similar to MIL-100(Cr), its extremely large specific surface area provides more adsorption sites for water molecules, nearly doubling the maximum saturation capacity compared to MIL-100(Cr). Its adsorption inflection point is slightly shifted to a higher relative pressure (about 40% RH), reaching a value close to its maximum capacity (about 1.1 g g^−1^(MOFs)) by 50% RH (Fig. 7).^88,89^

**Fig. 8:**
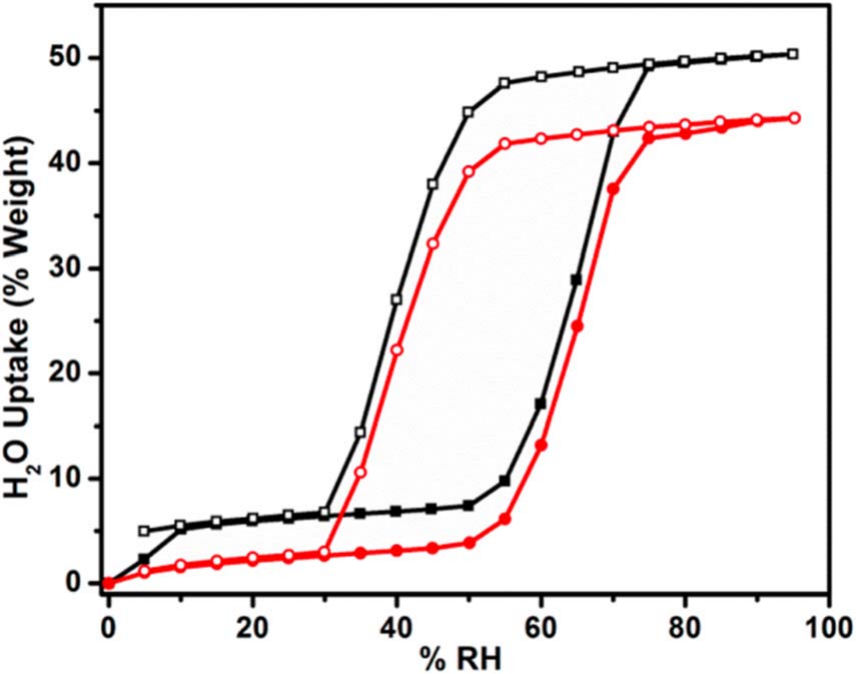
Water adsorption isotherms of Y-shp-MOF-5, with red curves measured at 25 °C and black curves measured after activation at 125 °C, reproduced from ref. 98 with permission from American Chemical Society,^98^ copyright 2017.

Hydrophobic mesoporous MOFs exhibit negligible water uptake in the low-humidity region. However, their typically larger pore sizes allow them to achieve measurable adsorption at high humidity almost exclusively through capillary condensation. The organic linkers in PIZOF-2(Zr) confer strong hydrophobicity, resulting in minimal adsorption at low humidity. Due to its mesoporous pores, a steep, step-like water vapor uptake begins at a high relative pressure (around 70% RH), approaching its maximum saturation capacity by approximately 80% RH. This abrupt adsorption behavior in the high-humidity region, caused by capillary condensation, leads to a significant hysteresis loop in PIZOF-2(Zr) (See its adsorption isotherm in Fig. 3).^13,62^ A similar adsorption profile is observed for Y-shp-MOF-5. Its primary adsorption inflection point is located near 60% RH, at the upper end of the medium humidity range. However, even after full activation, its CUS retain some water adsorption capability, resulting in a non-zero uptake at 10% RH. The isotherm plateaus between 10% and 55% RH, with capillary condensation again dominating the high-humidity uptake and giving rise to a hysteresis loop (Fig. 8).^95^

The mesoporous size range is quite broad. Some materials with pore sizes at the lower end of this spectrum can exhibit adsorption characteristics reminiscent of microporous MOFs, often showing their steep uptake step at lower RH compared to other mesoporous materials.

Ni-BPP and Ni-TPP are examples of materials with relatively small pores within the mesoporous range. They exhibit a step-like adsorption onset in the low-humidity region, although the steepness of the curve is less pronounced. Their isotherms represent a hybrid form combining features of Type I and Type V shapes. With moderate specific surface area and pore volume, they achieve a high maximum saturation water uptake of 0.8 g g^−1^(MOFs).^85^

**Fig. 9:**
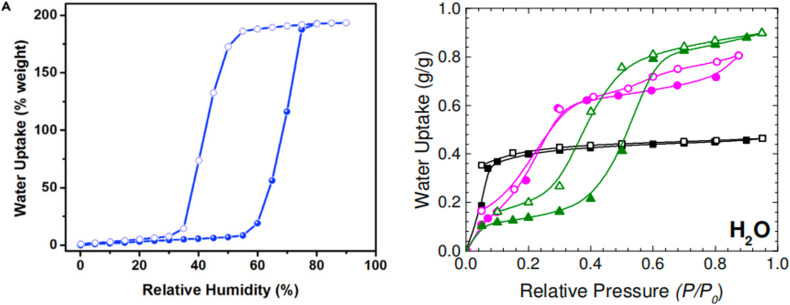
Water adsorption isotherms of Cr-soc-MOF-1 (left), reproduced from ref. 93 with permission from Elsevier,^93^ copyright 2017; and of Ni-MOF-74 (black), Ni-BPP (pink), and Ni-TPP (green) (right), reproduced from ref. 88 with permission from American Chemical Society,^88^ copyright 2017.

However, Cr-soc-MOF-1 does not follow this trend. It possesses record-high values for specific surface area and pore volume, yet features a relatively small pore size within the mesoporous range. Its S-shaped isotherm exhibits a delayed adsorption inflection point, located in the latter part of the medium humidity range. The steep uptake commences only at around 55% RH, reaching a remarkable water vapor adsorption capacity of 1.8 g g^−1^(MOFs) by 70% RH (Fig. 9).^90^

**Fig. 10:**
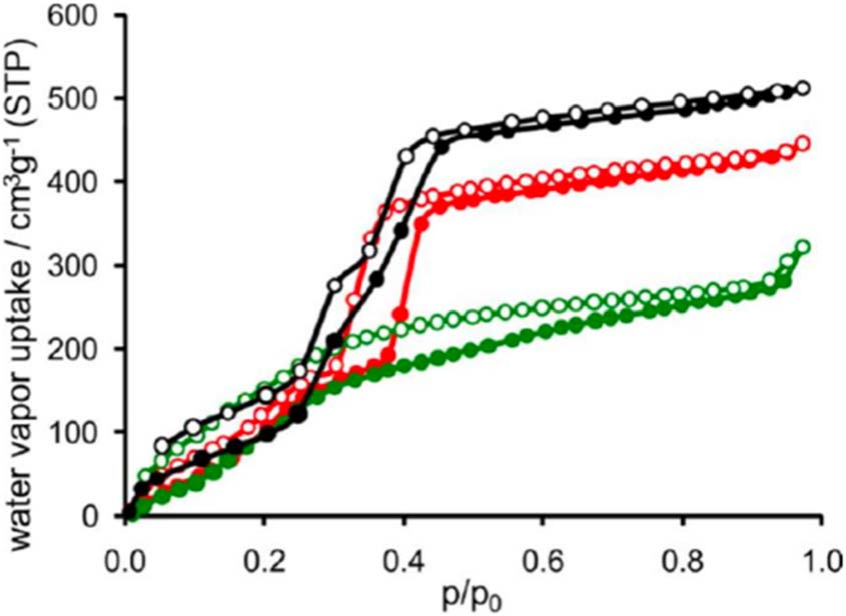
Water adsorption isotherms of the DUT series, with DUT-67 (black), DUT-68 (red), and DUT-69 (green), reproduced from ref. 41 with permission from American Chemical Society,^41^ copyright 2013.

The behavior of mesoporous MOFs with multiple pore sizes is similar to that of microporous MOFs, in that each pore size exhibits characteristic adsorption behavior. As mentioned above, the MIL-100 series contains a certain proportion of micropores, resulting in step-wise increases on the isotherms. Similarly, DUT-68(Zr) is a MOF with multiple pore sizes, featuring four distinct pore diameters ranging from the smallest micropore (0.8 nm) to mesopores (2.8 nm). It exhibits a step-wise adsorption starting at 40% RH and completes the step-wise process at closely spaced RH. Unlike many MOFs showing typical S-shaped isotherms, DUT-68(Zr) maintains a certain adsorption capacity at low humidity, which can be attributed to the filling of its four distinct pore sizes (Fig. 10). Similar to the dual-pore MIL-100 (Fe, Al), pores of different sizes are filled sequentially; however, the step-wise adsorption for each pore size is not pronounced. Several pores biased toward micropore dimensions ensure adsorption capacity at low humidity. The larger mesopores are primarily filled *via* capillary condensation, leading to pronounced hysteresis.^37,41,86^

Strictly speaking, genuinely macroporous (>50 nm) MOFs are relatively scarce compared to their more readily hydrophilic microporous and mesoporous counterparts, and research on water vapor adsorption in macroporous MOFs is even more limited. This scarcity stems from the intrinsic structural characteristics of MOFs: their frameworks consist of organic linkers and metal nodes. Larger pores necessitate longer linkers, which often compromise structural stability, increasing the risk of framework collapse and posing challenges in achieving crystallinity and stability during synthesis.^5,12^

Consequently, research on water vapor adsorption in pure macroporous MOFs remains a gap in the literature. Existing studies are primarily limited to composite materials incorporating MOFs with other components to form macroporous structures. For instance, Lu *et al.* fabricated a composite material using MOF-801, creating composite membranes with pores up to 6 nm—considered very large within the context of MOFs.^102^ However, its water vapor adsorption performance was poor. The large pore size resulted in a looser pore structure, a reduced number of capillary pores, and inefficient mass transfer, ultimately leading to diminished adsorption capacity.^102^ In another study, Yang developed a macroporous composite material using MOF-801 for atmospheric water harvesting. Under adsorption conditions of 20% RH and 25 °C and desorption at 85 °C, it achieved a water collection rate of 1.60 kg kg^−1^(composite) per day.^103^ However, this study did not characterize the specific physical properties of the material nor provide detailed water vapor adsorption isotherm data, focusing instead on the macroscopic pore structure and the practical performance in the application.^103^ This also underscores that macroporous MOFs are generally not well-suited for fundamental water vapor adsorption studies.

### 3.2 Specific surface area and pore volume

The specific surface area typically enhances water molecule uptake by increasing the number of available adsorption sites within the pores. A larger specific surface area means more sites are available on the internal surface to host water molecules. Consequently, macroscopic adsorption data often show that a larger specific surface area generally correlates with a higher water vapor adsorption capacity. Pore volume, on the other hand, increases the capacity for water uptake by providing more space within the pores to accommodate water molecules. Generally, specific surface area and pore volume are interdependent; a larger specific surface area is often accompanied by an increased pore volume. Together, they largely determine the material's maximum saturation uptake capacity. Pore size also influences the magnitude of both parameters, collectively dictating the water vapor adsorption behavior of the material.

**Fig. 11:**
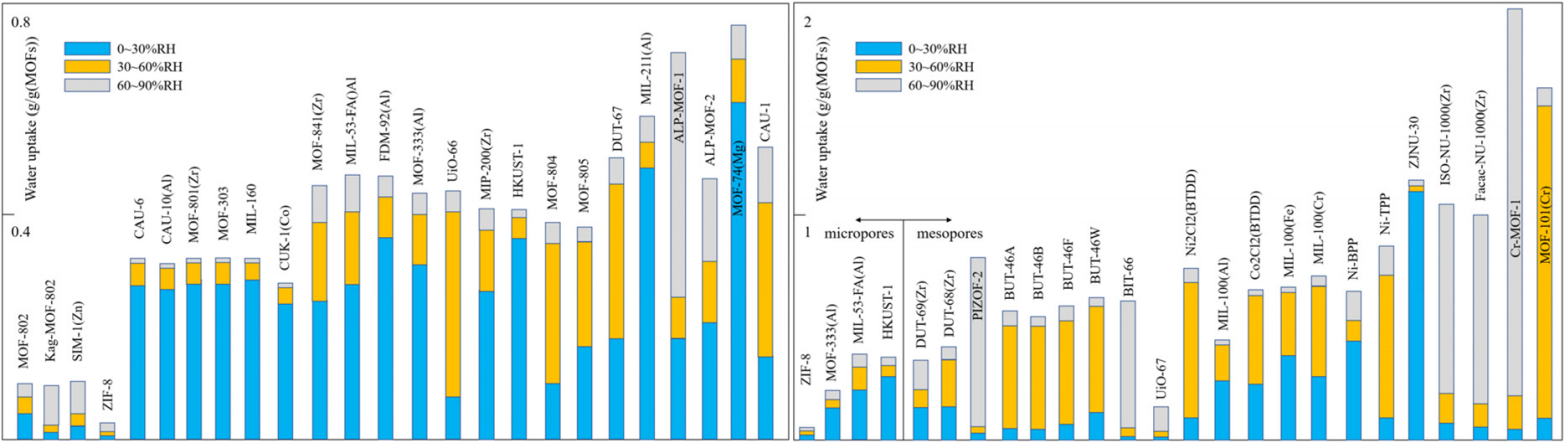
Water uptake of representative microporous(left)/mesopores(right) MOFs in various RH ranges; from left to right on the horizontal axis, the materials exhibit progressively increasing specific surface areas and pore volumes.

Microporous materials possess small pores, which typically result in a lower specific surface area and pore volume. After undergoing step-like water vapor adsorption in the low-humidity region, hydrophilic microporous materials approach their maximum saturation capacity at relatively low RH. Due to their limited specific surface area and pore volume, the pores lack sufficient space to hold more water molecules, leading to a generally low maximum saturation uptake (Fig. 11). CAU-6, with its relatively small pore size, specific surface area, and pore volume, exhibits some water adsorption capacity at low humidity (about 0.2 g g^−1^(MOFs) at 10% RH). However, after the initial adsorption on hydrophilic unsaturated sites, the uptake increases by only about 0.1 g by 90% RH, attributed to the limited pore space unable to accommodate additional water molecules at high humidity.^13^ This phenomenon is also observed in other microporous materials like CAU-10(Al) and MOF-801(Zr).^69,77^ Some microporous materials with larger pore sizes, such as CUK-1(Co, Mg, Ni), which have pores of nearly 1.5 nm, still exhibit a low maximum saturation uptake due to their even smaller specific surface area(around 500 m^2^ g^−1^) and pore volume (<0.3 cm^3^ g^−1^).^34^ The small pore size and hydrophilicity grant these materials some water adsorption capacity, but this is largely confined to the low-humidity region. At higher humidity, the limited specific surface area and pore volume prevent the pores from accommodating more water molecules, thus restricting the maximum uptake.

Nevertheless, some microporous materials possess a larger specific surface area and pore volume compared to those mentioned above. While their step-like adsorption still occurs at low relative pressures, they demonstrate a better maximum working capacity. In DUT-67, which features multiple pore sizes, the presence of a larger pore (1.6 nm) alongside the micropores (0.7 nm) increases the overall specific surface area and pore volume, resulting in a higher maximum water vapor uptake compared to other hydrophilic microporous materials. Similarly, the relatively large pore volume of MIP-211(Al) enables a maximum capacity of 0.6 g g^−1^(MOFs).^74^ This effect is more pronounced in ALP-MOF-1, which has a specific surface area of nearly 2000 m^2^ g^−1^, achieving a maximum saturation uptake of nearly 0.8 g g^−1^(MOFs). However, this trend is not distinctly observed in ALP-MOF-2, which, despite having a specific surface area comparable to ALP-MOF-1, does not achieve a proportionally matching adsorption capacity, although it still exceeds that of most microporous materials.^81^

This correlation between specific surface area/pore volume and uptake also applies to hydrophobic microporous materials. These materials not only lack sites for strong water interaction but their limited pore space further exacerbates the hydrophobic effect. SIM-1(Zn), with a specific surface area of 570 m^2^ g^−1^ and a pore volume of 0.3 cm^3^ g^−1^, exhibits a working capacity below 0.15 g g^−1^(MOFs).^45^ MOF-802 and MOF-2, with even smaller specific surface area and pore volume, have maximum uptake capacities of only around 0.1 g g^−1^(MOFs). Although ZIF-8 has a larger specific surface area and pore volume than the aforementioned materials, its water adsorption performance is poor, with a maximum saturation uptake of merely 0.02 g g^−1^(MOFs), indicating a stronger hydrophobic character.

Compared to microporous materials, the larger pore size of mesoporous MOFs offers greater potential for enhancing the specific surface area and pore volume of the pore network. It is notable that most mesoporous materials possess significantly superior pore space characteristics (larger specific surface area and pore volume) than their microporous counterparts, resulting in more ideal maximum adsorption capacities (Fig. 11). Among reported materials, MIL-101(Cr) and Cr-soc-MOF-1, which possess exceptionally high specific surface areas (up to nearly 6000 m^2^ g^−1^) and pore volumes (up to 2 cm^3^ g^−1^), demonstrate outstanding performance, achieving maximum water uptake capacities of nearly 2 g g^−1^(MOFs). Within the MIL-100 series based on Fe, Al, and Cr metal ions, which share comparable physical parameters and rank among MOFs with relatively large specific surface areas and pore volumes, the Cr and Fe variants exhibit the highest maximum uptake, while MIL-100(Al) shows the lowest. Although the precise specific surface areas for ISO-NU-1000(Zr) and Facac-NU-1000(Zr) were not characterized in the cited studies, their large pore volumes (as high as about 1.3 cm^3^ g^−1^) contribute to their considerable maximum adsorption capacities.^91,92^

Nevertheless, some mesoporous MOFs exhibit less ideal adsorption performance due to smaller structural parameters. DUT-68(Zr) and DUT-69(Zr) possess relatively small specific surface areas and pore volumes compared to most mesoporous materials (and even some microporous ones), leading to unimpressive maximum uptake. This is also true for some MOFs with multiple pore sizes; for instance, the significant presence of micropores in DUT-68(Zr) contributes to its low specific surface area and pore volume, limiting its maximum uptake to only about 0.3 g g^−1^(MOFs). UiO-67 represents an exception. Despite having physical parameters comparable to the MIL-100 series, its adsorption performance is poor,^93^ with a maximum capacity even lower than many microporous materials.

**Fig. 12:**
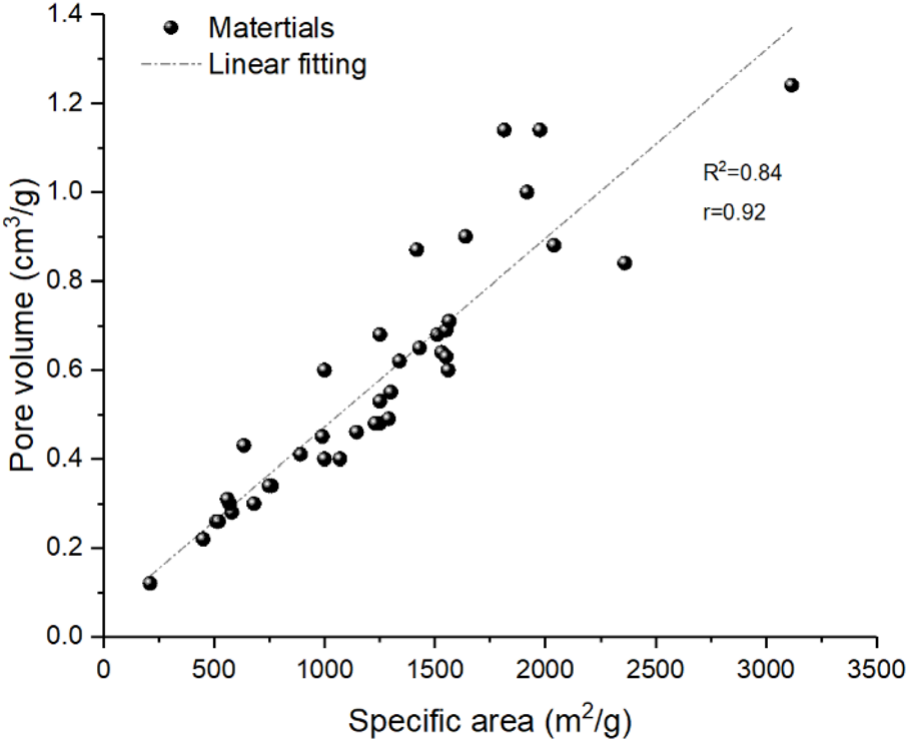
Correlation between pore volume and specific area.

**Fig. 13:**
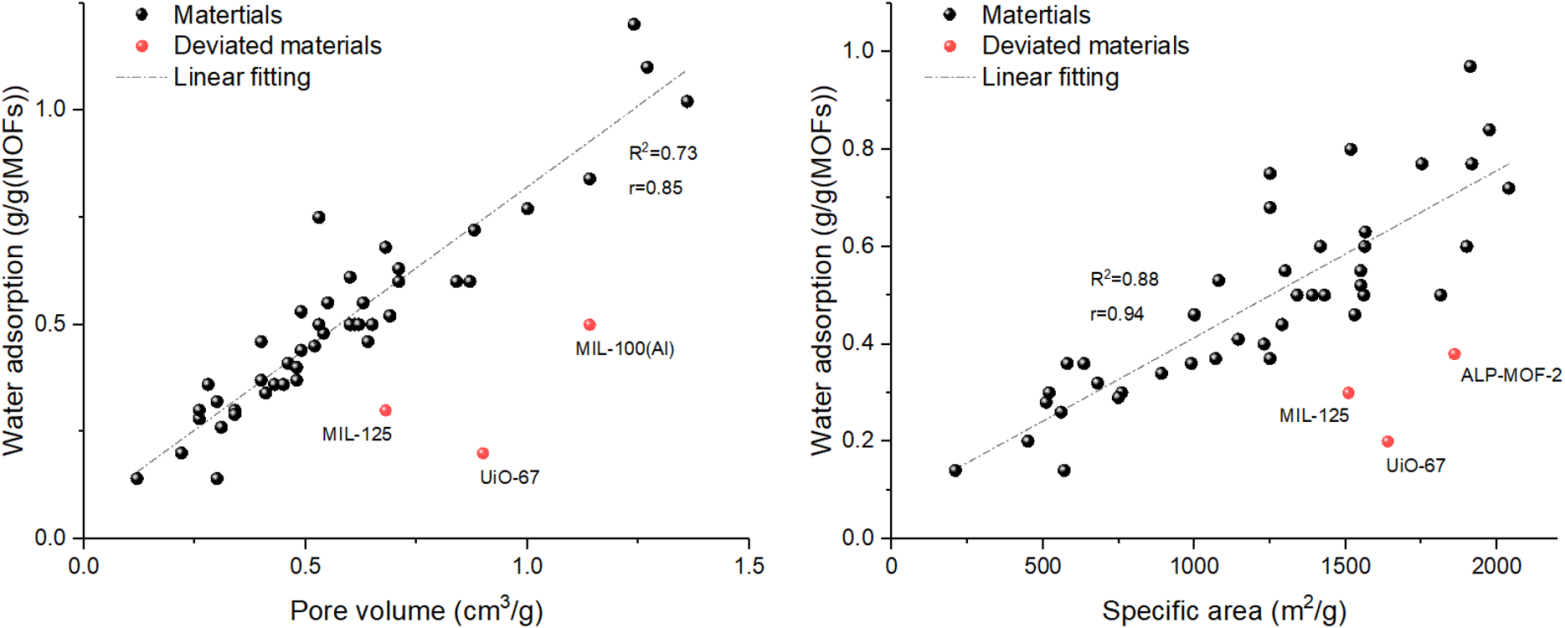
Correlation between pore volume(left)/specific area(right) and maximum water uptake for the MOFs.

To quantitatively examine the influence of specific surface area and pore volume on water adsorption capacity, we performed linear regression analyses using the data compiled in Table 1. As shown in Fig. 13, a strong positive correlation was observed between specific surface area and maximum water uptake, with a coefficient of determination *R*^2^ = 0.88 (Pearson *r* = 0.94). Similarly, pore volume exhibited a positive correlation with uptake (*R*^2^ = 0.73, *r* = 0.85). These results confirm that both parameters are important predictors of adsorption capacity, with specific surface area showing slightly stronger explanatory power in this dataset (Fig. 12).

Importantly, specific surface area and pore volume themselves are strongly correlated (*R*^2^ = 0.84, *r* = 0.92, Fig. 12). This inherent interdependence accounts for their similar predictive performance observed in Fig. 13.

The data scatter in all three plots underscores that while larger surface areas and pore volumes generally lead to higher water uptake, other factors—such as pore chemistry, hydrophilicity, and metal node characteristics—also play critical roles. For instance, certain materials deviate from the overall trend: UiO-67 exhibits a relatively high specific surface area (1639 m^2^ g^−1^) and pore volume (0.9 cm^3^ g^−1^) but a low water uptake (0.2 g g^−1^), likely due to its hydrophobic pore environment; MIL-125 (1510 m^2^ g^−1^, 0.68 cm^3^ g^−1^, 0.3 g g^−1^) and ALP-MOF-2 (1860 m^2^ g^−1^, 0.38 g g^−1^) also show lower-than-expected uptake. Conversely, MIL-100(Al) (1814 m^2^ g^−1^, 1.14 cm^3^ g^−1^, 0.5 g g^−1^) exhibit moderate deviations. These examples highlight the importance of considering pore chemistry and framework properties beyond simple physical parameters, as discussed in Section 3.3.

### 3.3 Influence of SBU geometry and metal type on water adsorption

Beyond pore size and surface area, the geometry of secondary building units (SBUs) and the nature of metal nodes play crucial roles in determining water adsorption behavior.

Zero-dimensional (0D) clusters, such as Zr_6_ and Cr_3_ nodes, typically form cage-like or three-dimensional pore networks.^104^ The high connectivity of Zr_6_ nodes (up to 12) enables exceptional structural diversity and stability, as exemplified by the **fcu**, **csq**, and **scu** topologies.^105^ Water adsorption in these MOFs is primarily governed by ligand design, with the metal clusters providing robust frameworks that withstand multiple adsorption–desorption cycles.^106,107^ Cr_3_-based MOFs (*e.g.*, MIL-101, Cr-soc-MOF-1) exhibit remarkable stability due to the kinetic inertness of Cr-carboxylate bonds, achieving ultra-high water uptakes up to 1.95 g g^−1^.^108–110^

One-dimensional (1D) chain-like SBUs, particularly those based on Al^3+^, represent a distinct class of water adsorbents.^111^ The *cis*–*trans* alternating AlO_6_ octahedral chains in MOF-303 and CAU-10 create hydrophilic 1D channels with diameters of approximately 6–8 Å.^112–114^ This unique pore geometry enables steep water uptake at low relative humidity (10–20% RH) with minimal hysteresis, making these materials ideal for atmospheric water harvesting in arid regions.^115^ Notably, CAU-10-H retains its adsorption capacity after 10 000 cycles, demonstrating exceptional stability.^112,113^

Regarding metal type, a general grouping can be established based on adsorption characteristics: high-valent metals (Zr^4+^, Cr^3+^, Al^3+^, Ti^4+^) form strong coordination bonds with carboxylate linkers, ensuring hydrothermal stability.^107,116^ Among these, Al^3+^-based MOFs exhibit a unique combination of low-humidity responsiveness and rapid regeneration due to their 1D channel structures.^111,115^ Divalent metals (Zn^2+^, Cu^2+^, Ni^2+^, Co^2+^) typically require azolate linkers to achieve adequate stability. While they can provide open metal sites for strong water interactions, their long-term stability under humid conditions requires careful evaluation.^116^

Among the various metal types, Al(iii)-based MOFs exhibit particularly favorable chemistry for water vapor adsorption. This superiority stems from three factors: (i) SBU geometry: Al^3+^ ions tend to form one-dimensional (1D) chain-like secondary building units, such as the *cis*–*trans* alternating AlO_6_ octahedral chains in MOF-303 and CAU-10.^111,112,114^ These chains create hydrophilic 1D channels with diameters of approximately 6–8 Å, which are optimally sized for water capture at low humidity^117^.(ii) Pore chemistry: the channel walls are lined with polar Al–O bonds and functional groups from organic linkers, providing strong affinity for water molecules without excessive binding strength that would hinder regeneration^118,119^.(iii) Adsorption behavior: due to this unique combination of pore geometry and chemistry, Al-MOFs exhibit steep water uptake at low relative humidity (10–20% RH) with minimal hysteresis, making them ideal for atmospheric water harvesting in arid regions.^111^ For example, MOF-303 achieves 0.4 g g^−1^ uptake at 20% RH and can be regenerated at temperatures as low as 85 °C, while CAU-10-H retains its adsorption capacity after 10 000 cycles. Furthermore, MOF-303's 1D channels with a 6.5 Å cavity diameter contribute to its hydrophilic properties and reduced water evaporation enthalpy, making it suitable for solar steam generation.^112,114^

These characteristics position Al-MOFs as leading candidates for water harvesting applications, demonstrating that the choice of metal and the resulting SBU geometry are critical factors in designing MOFs for targeted water adsorption performance.

### 3.4 Water adsorption characteristics of MOFs

Pore size dictates the water vapor adsorption behavior of a material by influencing the dominant adsorption mechanism within the pores:

For micropores, the extremely small pore dimensions facilitate water molecule adsorption primarily *via* hydrophilic sites (*e.g.*, unsaturated metal centers) and physical interactions at very low relative humidities. Unlike capillary condensation, even a small number of water molecules within the confined pore channels can initiate these physicochemical processes. This initial uptake constitutes the majority of the total water adsorption in hydrophilic microporous materials. Consequently, these materials typically exhibit a very early, steep (often S-shaped) adsorption step. Following this step, little additional uptake occurs in the medium to high humidity ranges. Furthermore, the confined pore space inherently limits the specific surface area and pore volume, resulting in generally unexceptional maximum saturation capacities. Regarding desorption, microporous materials typically show little to no hysteresis, consistent with a primary adsorption mechanism based on site-specific interactions and monolayer-multilayer formation rather than pore filling *via* condensation.

Mesoporous materials, with their larger pores, possess greater internal space, leading to superior specific surface areas and pore volumes compared to microporous analogues. This generally translates to a trend of higher maximum saturation uptake capacities. The step-like adsorption in hydrophilic mesoporous MOFs occurs at higher relative humidities, typically within the medium humidity range. A significant hysteresis loop is commonly observed in these materials, largely attributable to the increasing dominance of capillary condensation within the mesopores as the primary adsorption mechanism at higher RH, which exhibits this hysteretic effect upon desorption.

Strictly speaking, genuinely macroporous MOFs are exceedingly rare due to intrinsic structural instability challenges. Their formation is synthetically difficult, and consequently, studies on their pure water vapor adsorption are virtually non-existent. Reported instances of “macroporous” structures in this context are limited to composite materials incorporating MOFs, where the macropores are part of a secondary support structure. Therefore, fundamental research on water vapor adsorption in pure, well-defined macroporous MOFs remains a significant gap in the field.

## 4. Application of water adsorption in MOFs

Numerous MOFs demonstrate excellent performance not only in laboratory settings but also in practical applications. Among the applications centered on water vapor adsorption, atmospheric water harvesting and indoor dehumidification are the most extensively studied, with other uses such as thermal transformation systems or water delivery systems also receiving some scholarly attention. Different practical applications demand distinct water vapor adsorption characteristics from MOFs. For instance, in atmospheric water harvesting, which aims to collect moisture *via* adsorption in harsh, low-humidity climates, materials are required to exhibit a steep uptake step precisely within the low-humidity range and facilitate easy desorption. Conversely, for dehumidification purposes, maintaining indoor air humidity at a desired setpoint often necessitates materials that undergo a stepwise adsorption transition within the medium humidity range, accompanied by a pronounced hysteresis loop. This ensures that a passive device can rely solely on the material's intrinsic adsorption properties to maintain the required humidity level without external energy input. Similar, specific material requirements are evident across different applications. The following section will explore and summarize the requisite material properties for these common applications.

### 4.1 Atmospheric water harvesting

Water resources are fundamental to life and crucial for human survival and ecosystem maintenance. The atmosphere holds a substantial amount of water vapor, originating from the evaporation of Earth's vast water reserves, distributed even in the air over inland and arid regions. Significant research efforts have been devoted to atmospheric water harvesting.^120,121^ Common methods for capturing water vapor from air include cooling-condensation dew formation, fog collection, membrane-based collection, and adsorption.^122^ The adsorption method is a relatively efficient and straightforward approach for moisture capture, with less stringent environmental requirements. However, a key challenge lies in the subsequent desorption of water from the material, a process that accounts for the majority of the energy consumption, which is not insignificant.^123^

The material requirements for adsorptive atmospheric water harvesting can be summarized as follows: (i) a suitable isotherm shape, featuring a steep adsorption step within the low-humidity range; (ii) a high working capacity, encompassing not only a large saturation uptake but also a high amount of cyclable water released over multiple adsorption–desorption cycles; (iii) a low regeneration temperature, enabling water release from the material using minimal, low-grade thermal energy, even in harsh environments.^124,125^ These three points are the most critical. Of course, other important, though less pivotal, requirements exist, such as material stability determining longevity, rapid adsorption–desorption kinetics, and suitability for integration into devices. Based on these requirements, researchers have developed various water harvesting devices utilizing MOFs possessing such adsorption properties, conducting tests in real arid environments and achieving promising results.

**Fig. 14:**
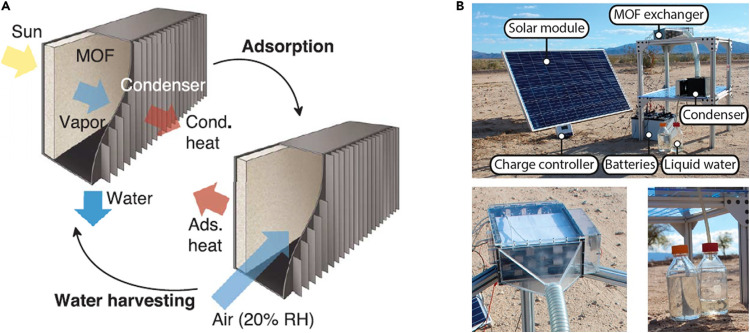
(A) Schematic illustration of a passive water-harvesting device from Kim *et al.*^6^; (B) photograph of an active water-harvesting device based on MOF-303 from Hanikel *et al.*,^65^ reproduced from ref. 26 with permission from Elsevier,^26^ copyright 2023.

Kim *et al.* compared the water vapor adsorption characteristics of MOF-801, UiO-66, and other MOFs, concluding that MOF-801 is suitable for arid regions with RH below 20%, while UiO-66 is better for higher humidity areas. They employed MOF-801 as the adsorbent in a passive water harvesting device. MOF-801's specific advantages make it particularly suitable for this application: a sharp uptake increase within a narrow, low RH window; low regeneration temperature; excellent stability and cycling performance; and low cost. The device, subjected to daily temperature cycles between 25–65 °C, collected 0.25 g g^−1^(MOFs) of water under ambient temperature and pressure at low humidity (20% RH). After structural optimization of the device, the daily water collection amount could be increased nearly tenfold.^6^ This harvesting method is entirely passive, relying solely on the material's high adsorption capacity, low desorption energy, and ambient conditions (Fig. 14).

Beyond passive systems, active devices using MOFs for adsorptive atmospheric water harvesting also exist. These are not limited to single daily adsorption-night/desorption-day cycles. For instance, one study leveraged the fast dynamic water vapor adsorption properties of MOF-303 to perform multiple adsorption–desorption cycles per day. This multi-cycling was achieved by incorporating forced convection and a temperature swing within the device to provide the necessary conditions for MOF-303. MOF-303 possesses not only a steep low-humidity inflection point and high working capacity but, crucially, its superior dynamic performance enables such active devices. The study reported that the water harvester could achieve a water production rate of approximately 0.7 g g^−1^(MOFs) per day under Mojave Desert conditions (27 °C, <10% RH) and 1.3 g g^−1^(MOFs) per day in an indoor arid setting (27 °C, 32% RH), demonstrating significant superiority over most passive devices.^65^

For atmospheric water harvesting, the paramount requirement for a material is its ability to exhibit a stepwise (steep) increase in water vapor uptake at low RH. Whether in passive or active devices, most systems discussed herein employ microporous MOFs as the adsorbent material. This preference stems from the fact that hydrophilic microporous MOFs not only offer a substantial working capacity but, most critically, provide an ideal adsorption response in the low-humidity region: they undergo their primary adsorption step within the 10%∼20% RH range, making them suitable for operation in harsh, arid conditions. The strength of their hydrophilic sites is typically sufficient for capture yet not excessively strong to hinder desorption. Furthermore, the general absence of a significant hysteresis loop in their water adsorption isotherms simplifies the water release process, as it avoids the need for more extreme conditions (*e.g.*, higher temperatures or lower pressures) for complete desorption.^51,126^

### 4.2 Indoor humidity control

Modern lifestyles have largely shifted from open outdoor living spaces to more secure and enclosed environments. To ensure comfort within these indoor spaces, most are equipped with air conditioning systems, which account for a substantial portion of energy consumption—over 40% of total building energy use.^3^ Traditional air conditioning humidity control relies on cooling-based dehumidification. This method removes both sensible and latent heat simultaneously. However, achieving the low dew point temperatures required for latent heat (moisture) removal leads to significant energy waste.^127,128^ A key issue lies in the independent control of indoor air temperature and humidity.^129,130^ Consequently, adsorption-based air conditioning systems have emerged. These systems utilize solid adsorbents to dehumidify air independently, potentially reducing the energy required to achieve target indoor humidity conditions. Nevertheless, the energy consumption for regenerating the solid adsorbent remains considerable, posing a limitation to the widespread adoption of solid desiccant cooling systems.^23,43,131^ In recent years, MOFs have emerged as promising alternatives to traditional desiccants, particularly due to their superior adsorption performance, high tunability, and relatively mild regeneration conditions.^8^

**Fig. 15:**
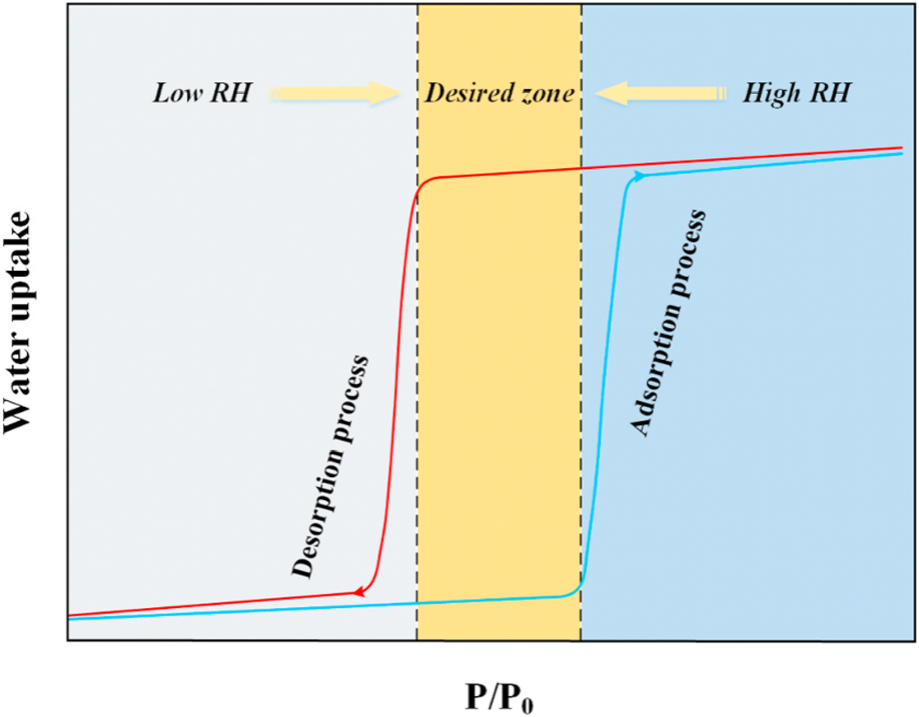
Ideal water adsorption isotherm required for MOFs applied in passive dehumidification systems, reproduced from ref. 3 with permission from Elsevier,^3^ copyright 2020.

The requirements for solid adsorbent materials applied in indoor dehumidification generally vary depending on the type of solid desiccant system. For passive dehumidification systems, materials are typically required to exhibit a steep water vapor adsorption step within the operational humidity range (American Society of Heating, Refrigerating and Air-Conditioning Engineers recommend comfort zone between 45%∼65% RH),^132^ accompanied by a distinct hysteresis loop within this range. They should also not be highly temperature-sensitive, maintaining a consistent isotherm shape across a reasonable temperature range (Fig. 15).^133,134^ For active systems, however, priority is given to materials that deliver a high cyclic working capacity within their steep adsorption transition region and exhibit little to no hysteresis loop within the operational humidity range.^23,73,86^ Although MOFs generally demonstrate better water vapor adsorption performance and milder regeneration conditions, those currently available still do not strictly meet all the aforementioned requirements for dehumidification applications. Furthermore, research focus remains predominantly on laboratory-scale material performance rather than real-world engineering integration. In practice, the overall dehumidification effectiveness of a solid desiccant system is determined not only by material properties but also by the specific system type and its operational demands. The following section will review some typical MOFs that have been applied in dehumidification systems or show potential for such applications.

**Fig. 16:**
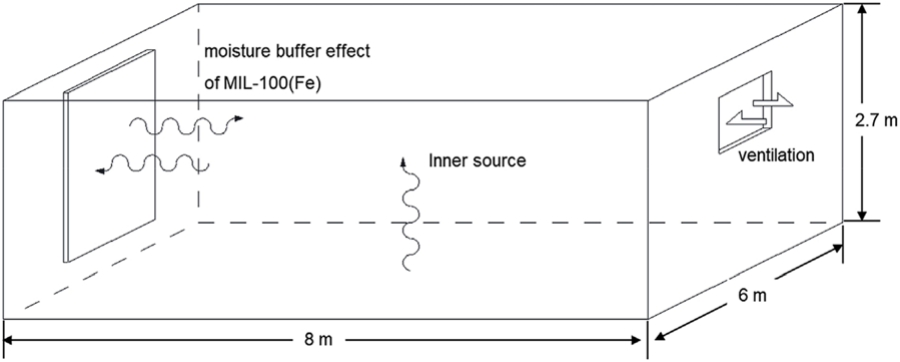
Application of MIL-100(Fe)-filled wall structures for indoor humidity control, reproduced from ref. 137 with permission from Elsevier,^137^ copyright 2018.

The MIL-100 series is among the most widely studied materials for water adsorption applications, with Al, Fe, and Cr commonly used as the metal ions. Materials in this series generally exhibit a steep water vapor adsorption step within the medium humidity range and possess considerable uptake capacity. Feng *et al.* proposed using MIL-100(Fe) as a passive humidity buffer in buildings, noting that its steep adsorption region aligns well with the indoor thermal comfort zone, demonstrating exceptional humidity buffering capability. The presence of a distinct hysteresis loop and good water stability further support its potential for use in passive dehumidification devices. Through simulation, they demonstrated that integrating MIL-100(Fe) into wall fillings could remove a significant portion of the latent heat load. Additionally, the ability of MIL-100(Fe) to be regenerated using low-grade heat further reduces system energy consumption (Fig. 16).^135^ MIL-100(Cr) exhibits water vapor adsorption behavior very similar to MIL-100(Fe). However, its practical application is limited due to the toxicity of Cr ions.^16,136^ MIL-101(Cr) offers a significant advantage over the MIL-100 series in terms of maximum saturation capacity, coupled with faster adsorption kinetics and robust water stability. Despite the continued use of toxic Cr, its ideal water vapor adsorption characteristics maintain its strong potential as a dehumidification material.^89^ Notably, Cr-soc-MOF-1 surpasses MIL-101(Cr) in specific surface area and pore volume, yielding the highest reported water vapor saturation uptake capacity to date. Its steep adsorption step located in the medium humidity range, coupled with a more pronounced hysteresis loop and high water stability, makes it a highly promising candidate for indoor humidity control applications.^90^

For indoor dehumidification applications, the most critical requirement for a material is its ability to exhibit stepwise (steep) water vapor adsorption within the medium-humidity range, coupled with a pronounced hysteresis loop. Most of the MOFs discussed above that meet this criterion are mesoporous materials. These adsorbents primarily rely on a combination of CUS and capillary condensation as the dominant adsorption mechanisms. Their characteristic adsorption profile in the medium-humidity region and the associated hysteresis loop ideally satisfy the material requirements for indoor humidity control. Furthermore, their typically large specific surface areas and pore volumes result in higher water vapor saturation capacities, making them more suitable for fulfilling the demands of practical applications.^90^

### 4.3 Other applications

MOFs are also applicable to numerous other practical fields leveraging their water vapor adsorption properties, such as adsorptive heat transformation, gas purification, and drying. These applications exploit the distinctive water vapor uptake behavior of MOFs and their selective adsorption towards various gases.

For instance, in adsorptive heat transformation, ideal MOFs are required to possess high hydrophilicity to meet adsorption demands in cooling applications, low regeneration temperature to reduce energy consumption during desorption, fast adsorption–desorption kinetics to enhance system efficiency, and excellent cyclic stability to achieve a high Coefficient of Performance.^71,137^

In contrast, for gas purification and drying applications, researchers utilize the selective adsorption capabilities of MOFs to separate components in gas mixtures (*e.g.*, gas drying), such as removing water from natural gas, dehydrating gas streams for storage, or drying inlet air for fuel cells.^138^ The requirements for these applications, however, show a less pronounced preference for specific pore sizes; MOFs with pores across various size ranges can be suitable depending on the specific context.

## 5. Summary and outlook

MOFs have emerged as highly promising adsorbents for water vapor adsorption-related applications, demonstrating significant advantages over traditional porous materials in terms of pore structure and water vapor adsorption characteristics. This review has highlighted the influence of key physical parameters—namely pore size, specific surface area, and pore volume—on the water vapor adsorption performance of MOFs. Furthermore, it has identified trends relating these structural parameters to the adsorption properties required in common practical applications:

1 Regarding pore size, hydrophilic microporous MOFs generally exhibit a steep, stepwise adsorption onset at low RH in their water vapor isotherms. This step is primarily driven by adsorption onto unsaturated hydrophilic sites or *via* molecular clustering, and is typically associated with minimal or no hysteresis. In contrast, hydrophobic microporous MOFs show flat isotherms or may display a steep uptake step only at very high humidity due to capillary condensation. However, the inherently low specific surface area and pore volume of microporous materials limit their pore space, resulting in generally modest maximum saturation water uptake capacities, even for those with high hydrophilicity.

2 Hydrophilic mesoporous MOFs typically feature a steep adsorption step within the medium humidity range. While contributions from site-specific adsorption and physical interactions persist, capillary condensation becomes increasingly dominant as the pore size increases. A pronounced hysteresis loop is commonly observed in these materials. Their larger specific surface areas and pore volumes, exceptionally high in some cases, lead to maximum saturation capacities that are significantly superior to those of microporous MOFs.

3 Specific surface area and pore volume are decisive factors influencing the maximum water vapor uptake capacity of MOFs. Across both microporous and mesoporous materials, those with larger values for these parameters generally exhibit higher water adsorption. This trend is particularly evident when comparing microporous and mesoporous materials, with the latter typically achieving higher capacities due to their more expansive pore space.

4 In addition to pore-related parameters, the geometry of SBUs and the nature of metal nodes play pivotal roles in determining water adsorption behavior. Zero-dimensional clusters, such as Zr_6_ and Cr_3_ nodes, provide exceptional structural stability and enable diverse topologies (**fcu**, **csq**, **scu**), with water adsorption primarily governed by ligand design. One-dimensional chain-like SBUs, particularly those based on Al^3+^, create hydrophilic channels (6–8 Å) that facilitate steep water uptake at low relative humidity (10–20% RH) with minimal hysteresis, making them ideal for atmospheric water harvesting. Regarding metal type, high-valent ions (Zr^4+^, Cr^3+^, Al^3+^, Ti^4+^) form strong coordination bonds with carboxylate linkers, ensuring hydrothermal stability, while divalent metals (Zn^2+^, Cu^2+^, Ni^2+^, Co^2+^) often require azolate linkers for adequate stability. Among these, Al(iii)-based MOFs exhibit a unique combination of low-humidity responsiveness and rapid regeneration due to their favorable 1D channel structures and moderate Al–O bond strength.

5 The requirements of specific practical applications dictate preferences for certain pore structure trends. For atmospheric water harvesting, the critical need for a steep uptake step in arid, low-humidity conditions makes hydrophilic microporous MOFs the primary candidates, provided they also offer sufficient working capacity over multiple cycles. Some mesoporous MOFs may also be feasible. Conversely, for indoor dehumidification, particularly in passive systems, materials must exhibit a steep adsorption step and a distinct hysteresis loop within the comfort humidity range—characteristics inherent to many mesoporous MOFs. Other applications impose specific requirements regarding adsorption energy, selectivity, hydrothermal stability, and cycling performance, though these do not show a strong, exclusive preference for a particular pore size.

Building on these insights, the development of next-generation MOF water adsorbents demands a more systematic and goal-oriented approach. Based on the structure–property correlations established in this review, we propose the following design roadmap for optimizing MOFs toward targeted applications: define application-driven targets. The desired adsorption behavior dictates the required pore characteristics: for atmospheric water harvesting in arid regions: steep adsorption step at 10–20% RH, uptake >0.3 g g^−1^ at 20% RH, regeneration temperature <70 °C, minimal hysteresis. For indoor humidity control: adsorption step within 40–65% RH, pronounced hysteresis loop, working capacity >0.5 g g^−1^ per cycle. For adsorption cooling: S-shaped isotherm with inflection at 20–30% RH, high working capacity, fast kinetics, coefficient of performance (COP) > 0.7. Select appropriate SBU and metal type based on stability and adsorption requirements. For low-humidity applications: prioritize Al-based 1D chain MOFs (*e.g.*, MOF-303, CAU-10, MIL-160) due to their inherent hydrophilic channels and low-humidity responsiveness. For high-capacity, long-cycle-life applications: consider Cr_3_-based MOFs (*e.g.*, MIL-101, Cr-soc-MOF-1) for their exceptional stability and ultra-high uptake. For general-purpose applications requiring structural versatility: Zr_6_-based MOFs offer tunable topologies and robust stability. Optimize pore size through isoreticular chemistry. Adjust pore dimensions by ligand extension or contraction to target specific RH ranges. Molecular simulations can guide the selection of optimal pore size prior to synthesis. Maximize pore volume and surface area while maintaining structural integrity. Pursue ligand extension strategies that preserve framework stability, avoiding capillary-force-driven collapse observed in some expanded MOFs. Consider multivariate approaches that combine large pore volumes with functional groups that enhance water affinity. Fine-tune pore chemistry through functionalization and node modification. Introduce hydrophilic groups (–NH_2_, –OH) to shift the adsorption step to lower RH, or hydrophobic groups to shift it to higher RH. Employ node modification strategies (metal exchange, anion exchange, capping agents) to enhance stability and tune adsorption behavior. Validate and scale. Evaluate performance under realistic operating conditions (dynamic cycling, variable humidity/temperature) after optimizing material properties. Develop green, scalable synthesis routes (water-based synthesis, continuous flow production) to facilitate commercialization.

Beyond material design, several cross-cutting challenges require attention: adsorption kinetics: while the influence of pore structure is well understood, strategies to enhance kinetics—such as creating hierarchical pores or reducing crystal size—need systematic investigation. Shaping and integration: powdered MOFs must be shaped into practical forms (coatings, pellets, monoliths) without sacrificing performance. Direct coating of MOFs onto heat exchangers has shown promise and deserves further development. Techno-economic analysis: future research should include assessments of material cost, synthesis scalability, and lifecycle environmental impact to guide commercial adoption.

In summary, although significant progress has been made in understanding how physical characteristics govern water adsorption in MOFs, the field is now poised to move from descriptive correlations to predictive design. By following the application-driven roadmap outlined above, researchers can systematically develop MOFs that not only exhibit excellent laboratory-scale performance but also meet the practical demands of real-world applications.

## Conflicts of interest

The authors declare that they have not known competing financial interests or personal relationships that could have appeared to influence the work reported in this paper.

## Data Availability

No new data was generated as part of this review.
